# Using Network Science to Analyse Football Passing Networks: Dynamics, Space, Time, and the Multilayer Nature of the Game

**DOI:** 10.3389/fpsyg.2018.01900

**Published:** 2018-10-08

**Authors:** Javier M. Buldú, Javier Busquets, Johann H. Martínez, José L. Herrera-Diestra, Ignacio Echegoyen, Javier Galeano, Jordi Luque

**Affiliations:** ^1^Laboratory of Biological Networks, Center for Biomedical Technology, Universidad Politécnica de Madrid, Madrid, Spain; ^2^Complex Systems Group and GISC, Universidad Rey Juan Carlos, Móstoles, Spain; ^3^Grupo Interdisciplinar de Sistemas Complejos, Madrid, Spain; ^4^ESADE Business School, Barcelona, Spain; ^5^INSERM-UM1127, Institute du Cerveau et de la Moelle Épinière. H. Salpêtrière, Paris, France; ^6^ICTP South American Institute for Fundamental Research, IFT-UNESP, São Paulo, Brazil; ^7^Grupo de Sistemas Complejos, Universidad Politécnica de Madrid, Madrid, Spain; ^8^Telefónica Research, Barcelona, Spain

**Keywords:** soccer, passing networks, network science, entropy, complexity, multilayer networks

## Introduction

During the last decade, *Network Science* has become one of the most active fields in applied physics and mathematics (Newman, 2010). From all its possible applications, in this Opinion paper we are concerned about the analysis of one of the most extended sports, football (soccer in U.S. terminology) (Sumpter, 2016), since it allows addressing different aspects of the team organization and performance not captured by classical analyses based on the performance of individual players. The reason behind relies on the complex nature of the game, which, paraphrasing the foundational paradigm of complexity sciences “*can not be analyzed by looking at its components (i.e., players) individually but, on the contrary, considering the system as a whole*” or, in the classical words of after-match interviews “*it's not just me, it's the team.”*

The recent ability of obtaining datasets of all events occurring during a match allows analysing and quantifying the behavior of a team as a whole, together with the role of each single player (Gudmundsson and Horton, 2017). Under this framework, the organization of a team can be considered as the result of the interaction between its players, creating passing *networks*, which are directed (i.e., links between players go in one direction), weighted (the weight of the links is based on the number of passes between players), spatially embedded (i.e., the Euclidean position of the ball and players is highly relevant) and time evolving (i.e., the network continuously changes its structure).

## Passing networks: information from a new perspective

**Figure 1:**
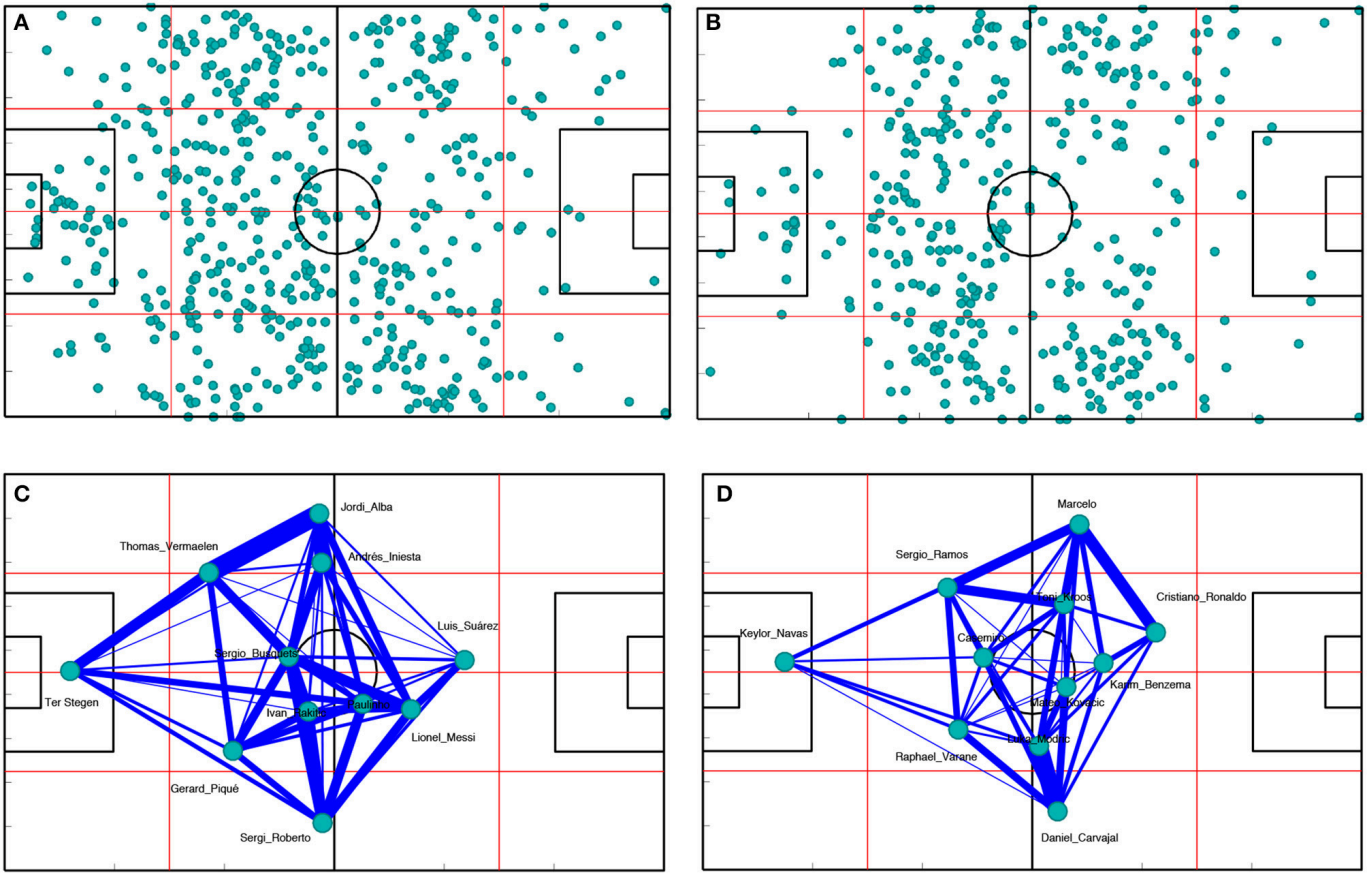
Construction of a passing network. In this example, passes from the match Real Madrid –Barcelona of the Spanish national league “*La Liga*”, season 2017/2018. In the upper row, initial position of all passes made by Barcelona **(A)** and Real Madrid **(B)**. In the bottom row, Barcelona **(C)** and Real Madrid **(D)** passing networks, where link widths are proportional to the number of passes between players, whose position in the network is given by the average position of all their passes. Datasets were provided by Opta and are accessible through the Supplementary Information.

Passes along the match give rise to three main types of passing networks: (i) *player passing networks*, where nodes are the players of a team (Passos et al., 2011; Grund, 2012), (ii) *pitch passing networks*, where nodes are specific regions of the field connected through passes made by players occupying them (Cintia et al., 2015) or (iii) *pitch-player passing networks*, where nodes are a combination of a player and its position at the moment of the pass (Cotta et al., 2013; Narizuka et al., 2014). See Figure 1 for an example of a player passing network.

Once the network is constructed, several “*topological scales”* can be identified inside the passing network of a football team: (i) the *microscale*, where the analysis is carried out at the level of nodes, i.e., the players and its role inside the network, (ii) the *mesoscale*, which ranges from small motifs describing the interaction between 3 or 4 players to the detection of larger groups of players that interact most frequently between them and (iii) the *macroscale*, which considers the network as a whole.

At the topological microscale, the importance of each player has been related to: its *degree*, which is the number of passes made by a player (Cotta et al., 2013); *eigenvector centrality*, a measure of importance obtained from the eigenvectors of the adjacency matrix (Cotta et al., 2013); *closeness*, measuring the minimum number of steps that the ball has to undergo from one player to reach any other in the team (López-Peña and Touchette, 2012); or *betweenness centrality*, which accounts how many times a given player is necessary for completing the routes (made by the ball) connecting any other two players of its team (Duch et al., 2010; López-Peña and Touchette, 2012). Other metrics, such as the *clustering coefficient*, which measures the number of “neighbors” of a player that also have passed the ball between them (i.e., the number of triangles around a player), has also been quantified to evaluate the contribution of a given player to the local robustness of the passing network (López-Peña and Touchette, 2012).

At the mesoscale level, the analysis of network motifs has shown how the overabundance of certain kinds of passes between groups of three/four players can be related to both the success of a team (Gyarmati et al., 2014) and the identification of leaders in the passing network (López Peña and Sánchez Navarro, 2015). Concerning the role of communities of players playing tightly connected between them, Clemente et al. (2015), related the high heterogeneity of the number of passes between players to the existence of sub-communities, which would hinder the behavior of the team as a whole. In the same sense, Gyarmati and Anguera (2015) studied all the recurring pass sequences, relating discovered sequence patterns to teams' playing style and strategy.

Finally, at the topological macroscale, a diversity of network metrics has been shown to be informative about the style and performance of football teams. For example, the position of the network centroid has been related to the performance of the teams (the more forward, the better) and has been shown to move backwards when teams play as visitors (Bialkowski et al., 2014). Other positional variables, such as the stretch index (mean dispersion of the players around the centroid), the surface area or the team length and width have also been used as more sophisticated metrics related to team performance (Duarte et al., 2012). On the other hand, Duch et al. (2010) designed a performance metric based on the betweenness of the players, showing how it correlated with the probability of winning a match. Other macro-scale measures such as the team average degree (i.e., average number of passes) or the variability of the players' degrees have also been proposed as proxies for evaluating team performance (Cintia et al., 2015; Pina et al., 2017). Concerning the macro-scale topology of the passing network, the small-world property (Watts and Strogatz, 1998), observed in a diversity of social, biological and technological networks, has also been reported (Narizuka et al., 2014). The average clustering coefficient of the team has also been shown to be much higher, during a match, than in equivalent random networks, unveiling the creation of triplets between players (Cotta et al., 2013). More recent studies tracking the position of the players have shown that it is better to maintain a balanced betweenness and a high closeness along the nodes of the passing network (Gonçalves et al., 2017).

Finally, it is worth mentioning that despite the micro- (i.e. player) and macro- (team) scales are perfectly identified, it is not clear how to define mesoscale units: What is the role of small motifs? Are there groups of players that can be considered as a unit? What is the role of small communities of players in the game? All these questions are of extreme importance and can be addressed using Network Science methodologies such as clustering or community identification algorithms (Fortunato, 2010), quantification of overrepresented motifs (Milo et al., 2002) or analysis of hierarchical structures (Corominas-Murtra et al., 2013).

## Some challenges: dynamics, space, time and interaction between teams

Passing networks are, in fact, dynamical system themselves, and the full identification and quantification of how variables determine the evolution of the game of a team are still open problems. Indeed, since the game cannot escape from the existence of stochastic forces combined with the high complexity of its intrinsic dynamics, modeling and forecasting a football match becomes a highly challenging task. Fortunately, distinguishing noise from determinism is an issue where Network Science can help, since it is possible to determine the *level of randomness* of the topology of the network and the dynamics occurring in it (e.g., how the ball moves along the network). We are on the way of constructing adequate null models of passing networks that are able to quantify the amount of disorder and complexity of the network topology. As explained in Sarzynska et al. (2016), the interpretation of network metrics should be referred to reference values, which can be obtained from adequate null models. However, these null models must incorporate the particular features of the system they are describing, and the Euclidean position of the nodes and temporal evolution should be taken into account (Sarzynska et al., 2016). Therefore, null models for passing networks must be as realistic as possible and include the intrinsic features of the game such as the degree distribution, length of the passes and positions of the players in the pitch. Concerning the dynamics along the network, recent approaches using Markovian models could be a starting point to unravel hidden patterns in the passing sequences of a team (López Peña, 2014) but must include the particular features of players' movements and their ability of decision.

Nonetheless, topology is only one dimension of the analysis of passing networks and at least other two must be included to have a complete picture: space and time. Concerning the former, the division of the pitch into different sub-regions has been carried out in a series of papers; however, it is not clear what is the most adequate spatial partition or, even if a unique partition (or scale) exists for a given team. From the division of the pitch into 6x3 rectangles of equal size up to a segmentation of 100 regions (Cintia et al., 2015) a diversity of field partitions has been suggested (Camerino et al., 2012; Narizuka et al., 2014; Arriaza-Ardiles et al., 2018). The translation of passes into pitch or pitch-player networks seems to be a promising complementary vision of the game, despite the information loss about the players' behavior.

Time is another dimension traditionally overlooked when constructing passing networks. Note that the analysis of passing networks must take into account its continuous evolution in time and space. For example, as shown in Duarte et al. (2012), entropy decreases as the game evolves, which is attributed to the fatigue of the players. In addition, collective behaviors decrease in complexity/irregularity during the time periods, accompanied with an increase of the deviations from the mean tendency. Network's density, heterogeneity and centralization also show differences between the 1st and 2nd part of a match (Clemente et al., 2015). However, it is common to average along the whole match (Gonçalves et al., 2017), or even along a competition (López-Peña and Touchette, 2012; Gyarmati et al., 2014), obtaining a network that can be informative about the general behavior of a team but excludes the unavoidable fluctuations during a match. Considering the networks during each of the two parts (Clemente et al., 2015) or constructing sliding windows of a certain length (between 5 and 15 min) are reasonable approaches (Yamamoto and Yokoyama, 2011; Duarte et al., 2012; Cotta et al., 2013) as suggested in Ribeiro et al. (2017). More recently, the construction of *temporal multilayer networks* has been proposed (Ramos et al., 2018). Under this framework, each temporal layer consists of a sub-network only containing the passes during a specific attacking phase, starting with the ball recovery and finishing with a shot or a ball loss. As explained in Ramos et al. (2018), aggregated or averaged networks contain unreal paths (or shortest-paths) between players, since these paths may go through passes that occurred at, for example, different parts of the match. Using temporal multilayer networks overcomes this issue, however, assuming an *a priori* temporal division of the network may introduce some bias into the network metrics and alternative approaches should focus on finding the time scales of the match from the observation of the game. This could be done by computing the network properties for any possible temporal division and, next, using community detection algorithms (Fortunato, 2010) to identify the optimal temporal partition of the match into time slots. Note that these time slots may have different lengths and would be indicators of how (and when) the game style changed, quantifying the temporal scales associated to each team.

At the same time, a football match is the result of the competition between two teams, i.e., the interaction between two networks. Therefore, the passing network of a team must be analyzed in combination with the network of the opponent (Narizuka et al., 2014). In this way, it will be possible to draw conclusions about how a team adapts its game depending on the opponent and what kind of topological organization leads to better results. Recent studies about *networks-of-networks* in other fields have shown that when networks get connected, important properties of the ensembled systems are modified (Boccaletti et al., 2014; Kivelä et al., 2014). With this regard, a multilayer description of a match, with two interacting networks, or layers, composed of the internal passes of each team, is still missing. This multilayer approach based on networks-of-networks would be complementary to the temporal multilayer networks proposed in Ramos et al. (2018). Specifically, the intra-layer links would be composed of the passes only within each team, while the inter-layer links would consist on ball recovery/losses. The analysis of the resulting network-of-networks could be fundamental to understand the evolution and adaptability of the teams along the match, which cannot be interpreted without analysing the behavior of each team separately. In this way, the competition between the two networks, suggests the application of the network-of-networks framework proposed in Aguirre et al. (2013), where it was shown that it is possible to find the optimal strategies of interaction with other networks, or in the case of football passing networks, that each team should find the most appropriate way of organizing its passing network according to the organization of the opponent.

On the other hand, there are parallel methodologies, such as the use of hypernetworks (Johnson, 2016), which would benefit from the inclusion of the information contained in the passing networks. Ramos et al. (2017) proposed the use of hypernetworks as an alternative way of studying the spatial competition and cooperation between players, translating actions occurring between two or more players into hypernodes. As they suggest, including passes between players in the hypernetwork seems a promising step forward in the application of hypernetworks to understand the organization of the game.

Finally, it is possible to translate and generalize the results of network theory in football to organizational studies (Orlikowski, 1996; Padgett and Powell, 2012). When two teams are competing in the field, they need to develop strategies to create new options and “entrepreneurial actions” that generate “surprises” to the opponent (Dew, 2009). Furthermore, the interaction between teams goes beyond the notion of “adaptation” challenging the concept of the “interface” or dynamic limit between the two teams. We believe that teams (beyond football and sports) need to generate new competencies such as systematic creativity and organizational learning that allow them to anticipate to the competition, promote their superiority and to create order and optimal organizational structures but, at the same time, to generate “disorder” in the opponent with the aim of creating situations of superiority.

## Author contributions

All authors participated in the conception of the article. JMB wrote the initial draft. All authors revised the manuscript together; all authors gave final approval for publication. Datasets were provided by Opta and are available through the Supplementary Information.

### Conflict of interest statement

The authors declare that the research was conducted in the absence of any commercial or financial relationships that could be construed as a potential conflict of interest.
